# Correction: The social amplification and attenuation of COVID-19 risk perception shaping mask wearing behavior: A longitudinal twitter analysis

**DOI:** 10.1371/journal.pone.0334033

**Published:** 2025-10-08

**Authors:** Suellen Hopfer, Emilia J. Fields, Yuwen Lu, Ganesh Ramakrishnan, Ted Grover, Qiushi Bai, Yicong Huang, Chen Li, Gloria Mark

The following grant number is missing from the Funding statement: NSF IIS-2107150.

The sixth author’s name is spelled incorrectly. The correct name is: Qiushi Bai. The correct citation is: Hopfer S, Fields EJ, Lu Y, Ramakrishnan G, Grover T, Bai Q, et al. (2021) The social amplification and attenuation of COVID-19 risk perception shaping mask wearing behavior: A longitudinal twitter analysis. PLoS ONE 16(9): e0257428. https://doi.org/10.1371/journal.pone.0257428.
