# Supplementary material for: The social amplification and attenuation of COVID-19 risk perception shaping mask wearing behavior: A longitudinal twitter analysis
Source: PLoS One. 2021 Sep 23;16(9):e0257428. doi: 10.1371/journal.pone.0257428 (PMC8460003; doi:10.1371/journal.pone.0257428)
Supplement: S1 List — (DOCX) [file pone.0257428.s001.docx]

**S1 List. List of keywords used to filter the dataset**

coronavirus
新型冠状病毒
防疫
方舱医院
武漢
coronavirusitalla
コロナ
coronaviruscanada
wuhan
신천지
koronawirus
coronarvirusitalia
冠状病毒
coronavirusupdate
新型冠狀病毒
novelcoronavirus
coronaoutbreak
新型肺炎
新冠
covid
coronavirus
italycoronavirus
武汉肺炎
武漢肺炎
2019冠状病毒
新型冠状病毒肺炎
武漢頑張れ
新冠肺炎
coronavirustruth
coronavirusitaly
新型コロナウイルス
covid19
chinapneumonia
covid-19
coronovirus
coronavirusaustralia
コロナウイルス
coronavirusoutbreak
covid19canada
koronavirus
코로나바이러스
武汉
코로나19
wuhanpneumonia
cdc
2019ncov
코로나
covid2019
ochca
coronavirusuk
covid19italia
方室病院
wuhancoronavirus
coronaviruschina
대구
方舱医院
