# Supplementary material for: The social amplification and attenuation of COVID-19 risk perception shaping mask wearing behavior: A longitudinal twitter analysis
Source: PLoS One. 2021 Sep 23;16(9):e0257428. doi: 10.1371/journal.pone.0257428 (PMC8460003; doi:10.1371/journal.pone.0257428)
Supplement: S2 List — (DOCX) [file pone.0257428.s002.docx]

**S2 List. List of 150 top news propagators filtered out of the dataset**

kr3at

TomthunkitsMind

Reuters

business

openletterbot

htTweets

SkyNews

thehill

CNN

timesofindia

globaltimesnews

COVIDLive

TPE_connect

EcoInternetDrGB

IndiaToday

ndtv

Independent

cnnphilippines

naija_reports

XHNews

AnalyticaGlobal

realTuckFrumper

ANI

CGTNOfficial

cnni

QuickTake

ABSCBNNews

threadreaderapp

PaperbirdsH

ABC

MailOnline

TimesNow

SCMPNews

DailyMirror

NBCNews

CNBC

jamesvgingerich

SafetyPinDaily

ANCALERTS

mitchellvii

guardian

SputnikInt

raybae689

En24_News

EBCNEWSs

washingtonpost

ChinaDaily

mapsofworld

nytimes

AFP

dev_discourse

airnewsalerts

AndyVermaut

badseedbook

VodafoneIN

republic

ChannelNewsAsia

latimes

Only_rock_radio

inquirerdotnet

PTI_News

mytentaran

samehelbarqy

rtehrani

rapplerdotcom

Apex_WW

disclosetv

AlArabiya_Eng

Jerusalem_Post

covid_19bot

bsindia

businessinsider

Loneprotester

bitcoinconnect

pennewstweet

WIONews

evankirstel

PneumoniaWuhan

STcom

RawStory

mlnangalama

CTVNews

gmanews

ErnstNordholt

nypost

DrCoronavirus

coronavid19_bot

MSNBC

ians_india

indiatvnews

WarsontheBrink

NorbertElekes

COVID19Masks

JoeFreedomLove

WakeUpCanada1

dailydigger19

florida_finesse

IndoPac_Info

511NY

BNODesk

CCTVAsiaPacific

Megaman512

techjunkiejh

HackneyedAbbort

airtelindia

the_hindu

ZyroFoxtrot

BloombergAsia

arabnews

Knewz_Currently

Airtel_Presence

RT_com

4PawShop

EndGameWW3

NewsbyMorB

PDChina

ASBreakingNews

newworldsurvive

WSJ

thefirstindia

Echinanews

DailyMailUK

Fxhedgers

TwitterLive

NYTHealth

IAM__Network

Crwth_

daily_trust

MusafirNamah

Bita33088889

LarryBoorstein

ZyiteGadgets

SocialistVoice

artgirl_andrea

firstpost

FCoronavirus19

Covid19Rtn

TheTop10News2

moneycontrolcom

Oneindia

CNNnews18

Moespi1

latestly

NewsTeq

Covid19Develop1

dcexaminer

PulpNews

ByCovid

DailyMail

nowthisnews
