# Supplementary material for: The social amplification and attenuation of COVID-19 risk perception shaping mask wearing behavior: A longitudinal twitter analysis
Source: PLoS One. 2021 Sep 23;16(9):e0257428. doi: 10.1371/journal.pone.0257428 (PMC8460003; doi:10.1371/journal.pone.0257428)
Supplement: S1 Table — (DOCX) [file pone.0257428.s003.docx]

**S3 Table. Codebook**

| Code | Description | Inclusion Criteria | Exclusion Criteria |
| --- | --- | --- | --- |
| Mask guidelines and policies | Describes tweets that discuss mask guidelines and policies as it relates to risk perceptions among Americans | Includes discussions about the health authorities who recommend guidelines (e.g. discussions about their credibility). Health authorities may be at the federal, state, or local level (e.g. CDC, US Surgeon General, Dr. Fauci, local health departments) | Excludes Trump as an authority who recommends guidelines. For these tweets, code under: Politics legitimizing risk |
|  |  | Includes discussions about the guidelines or references to the guidelines (e.g. CDC recommends that healthcare workers make cloth masks if they run out of PPE...) | Excludes discussions about political figures. For these tweets, code under: Politics legitimizing risk |
|  |  | Includes discussions that clarify guidelines or changes to the guidelines | Excludes discussions about mask mandates such as official orders or laws enforcing face masks. For these tweets, code under: Politics legitimizing risk |
|  |  | Includes discussions about mask-wearing policies (e.g. policies from corporations, schools, hospitals, etc.) | For tweets  discussing subgroups at risk as noted in the guidelines, double code with: Who is at risk? |
|  |  |  | For tweets discussing guidelines and referencing mask effectiveness, double code with: Mask Effectiveness |
| Political legitimizing of risk | Describes tweets that discuss politics as it relates to risk for Americans | Includes discussions about political ideologies | Excludes the police as government officials |
|  |  | Includes discussions about political figures (e.g. governors) and government systems at the federal, state, or local level | Excludes non-relevant references or mentions of political figures. |
|  |  | Includes discussions about risk and the government's handling of the pandemic (e.g. lack of mask manufacturing or coordination) | Excludes the mask wearing behavior of non-politicians. For these tweets, code under: Mask Behavior of Others |
|  |  | Includes discussions about mask mandates (i.e. official orders or laws enforcing face masks) |  |
|  |  | Includes tweets with relevant (i.e. risk-related) references or mentions of political figures |  |
|  |  | Includes direct observations, reactions, or comments about the mask wearing behavior of politicians |  |
|  |  | Includes calls or reminders made by political figures to wear a mask |  |
| COVID severity | Describes tweets that discuss social behaviors or reports that downplay or amplify risk | Discussions may occur via media reports, personal observations, etc. | Excludes tweets that discuss healthcare workers personally feeling at risk. For these tweets, code under: Who is at risk? |
|  |  | Includes discussions about behaviors related to severity (e.g. hoarding masks) | For tweets referencing the subgroup impacted by COVID, double code with Who is at Risk? |
|  |  | Includes discussions about changes in social behavior or operations as they relate (e.g. changes in travel or personal/social life) | Excludes only a (@) mention of a media source (e.g. @ABCNews) |
|  |  | Includes discussions about mask demand, costs, and shortages | For tweets that include healthcare workers reusing masks and being at risk, code under:  Who is at risk? |
|  |  | Includes discussions about cases, deaths, outbreaks, or hotspots |  |
|  |  | Includes discussions about other evidence of severity (e.g. sequelae, price gouging) |  |
|  |  | Includes comparisons with COVID. Comparisons may include racism, other known or unknown risk, or other diseases (e.g. flu, Ebola, HIV) |  |
|  |  | Includes discussions of companies or factories mass producing masks |  |
|  |  | Includes discussions about COVID hoaxes or conspiracies |  |
|  |  | Includes discussions about masks from China or Wuhan |  |
| Who is at risk? | Describes tweets that discuss personal risk or risk among subgroups | Includes assigning risk based on personal evidence (e.g. having to work during COVID or role as an essential worker) | For tweets discussing subgroups at risk as noted in the guidelines, double code with: Mask guidelines and policies. |
|  |  | Includes discussions about who should be wearing a mask to reduce risk | For tweets that generally call for everyone to wear a mask, code under Mask Behavior of Others |
|  |  | Subgroups may include: healthcare workers, essential workers, infected or sick individuals, elderly, high risk individuals, protesters, inmates, police, the general public, etc. "Everyone" is included as a subgroup in the context of discussing mask guidelines. Subgroups should be explicitly stated | Excludes donation tweets that do not mention risk |
|  |  | Includes discussions about donations to hospitals or other subgroups indicating they are at risk. NOTE: The words "in need" or "help" are sufficient for implied risk. | Excludes tweets about mask advertisements |
|  |  | Includes mentions of health care workers having to reuse masks | As stated in the inclusion criteria, this code can include the actions of others such as making masks. However, for tweets about the mask-wearing actions of others, code under Mask Behavior of Others |
|  |  | Includes discussions about explicit reasons for feeling at risk (e.g. susceptibility, health status, an essential worker, lack of vaccines, lacking proper PPE, etc.) | For statements about feeling at risk because of the mask behaviors of others,  code under "Mask Behavior of Others" |
|  |  | Includes discussions about explicit reasons for not feeling at risk or non-mask related behaviors that may reflect not feeling at risk (e.g. continuing with vacation plans despite COVID) |  |
|  |  | Includes discussions about seeking information about risk (e.g. Google) |  |
|  |  | Includes discussions about exchanging risk-related information with families, friends, and other acquaintances (e.g. warning others) |  |
|  |  | Includes tweets that discuss recommending or reinforcing mask behavior with families, friends, and other acquaintances (e.g. giving a mask) |  |
|  |  | Includes discussions about personal experiences with COVID (e.g. being positive or testing for COVID) |  |
|  |  | Includes discussions about personal networks with COVID |  |
|  |  | Includes mask wearing norms on a personal level |  |
|  |  | Includes discussions about masks as a way to reduce risk. Risk does not have to be explicitly stated. (e.g. wearing a mask or deciding to wear a mask, thinking about wearing a mask, wanting to wear a mask, looking for a mask, purchasing masks, making a mask, etc.). Can include the actions of others, however see exclusion criteria for details |  |
|  |  | Includes explicit statements about reasons why an individual wears a mask |  |
| Mask behavior of others | Describes tweets that discuss the mask behaviors of others in the context of risk (e.g. putting others at risk or collective efforts to reduce risk) | "Mask behaviors" include wearing a mask (e.g. role modeling), not wearing a mask, or improper mask wearing | Excludes discussions that express an attitude toward the mask behaviors of others in absence of risk |
|  |  | "Others" includes medical, scientific, or public health figures, journalists, celebrities, sports figures, police officers, protesters (e.g. BLM), the general public or those in a personal network | For tweets about others passing out masks, code under COVID Severity |
|  |  | Discussions may occur via direct observations, reactions, or comments | Excludes the mask wearing behavior of politicians. For these tweets, code under Political legitimizing of risk |
|  |  | Includes discussions related to racism, stigma, and the mask wearing behaviors of particular subgroups (e.g. Asians or Asian Americans) | For tweets mentioning whole subgroups wearing a masks, double code under Who is at Risk (e.g. "Clinicians wear masks") |
|  |  | Includes general calls or reminders from non-political figures to wear a mask or not to wear a mask |  |
|  |  | Includes mask wearing norms as they refer to the mask wearing behaviors of others or of a collective "everyone" |  |
|  |  | Includes hypothetical discussions about the mask behavior of others as well as potential consequences from the mask behavior of others (e.g. second wave, more deaths, putting others at risk, etc.) |  |
| Mask effectiveness | Describes tweets that discuss mask effectiveness in the context of risk | Includes discussions about different mask types and their effectiveness | Does not include other protective measures |
|  |  | Includes discussions about the credibility of a mask’s effectiveness or the risks associated with mask wearing (e.g. breathing in CO2) |  |
|  |  | Includes clarifying myths about masks and mask effectiveness (e.g. Masks protect others, not yourself) |  |
|  |  | Includes discussions about whether masks are needed or necessary |  |
| COVID desensitization | Describes tweets that discuss the sustainability of risk | Includes discussions about feeling tired, fed up, or not scared of COVID |  |
| Nonrelevant | Describes non-relevant tweets | Includes tweets not relevant to the current codes |  |
|  |  | Includes tweets that are unclear or difficult to understand |  |
|  |  | Includes tweets discussing other countries |  |
