# Supplementary material for: The social amplification and attenuation of COVID-19 risk perception shaping mask wearing behavior: A longitudinal twitter analysis
Source: PLoS One. 2021 Sep 23;16(9):e0257428. doi: 10.1371/journal.pone.0257428 (PMC8460003; doi:10.1371/journal.pone.0257428)
Supplement: Attachment — Submitted filename: ResponsetoReviewers.pdf [file pone.0257428.s004.pdf]

UNIVERSITY OF CALIFORNIA, IRVINE

BERKELEY • DAVIS • IRVINE • LOS ANGELES • MERCED • RIVERSIDE • SAN DIEGO • SAN FRANCISCO SANTA BARBARA • SANTA CRUZ

Program in Public Health
653 E. Peltason Drive
Anteater Instruction &
Research Bldg
Irvine, CA 92697-3957
FAX (949) 824-0529

August 15, 2021

Dear PLOS ONE Editors for Behavioral and Social Sciences/Public Health,

Attached is the manuscript revision entitled, "The social amplification and attenuation of COVID-19 risk perception shaping mask wearing behavior: A longitudinal Twitter analysis." A marked up and clean version are attached.

We thank the reviewers for their feedback and provide responses to reviewers' questions below:

R1: Discussion statements about implications seem unconnected to the actual analysis. This is true in many places but as one example, what part of the analysis justifies the claim that "an information deficit approach to public health messaging will unlikely be sufficient".

Response: Thank you for this comment. The implications section in the discussion section has been revised. Under the heading "implications for public health messaging" discussion has been edited to be grounded in the analysis of the study. The mask tweets expressed six aspects of COVID-19 risk perception that both amplify and attenuate COVID-19 risk perception. This complicates effective public health messaging around mask wearing. Results indicate that with the simultaneous amplification and attenuation of risk perception, that messages emphasizing structural determinants of risk e.g., workplace policies (and a shift away from individual focused messaging) may yield the highest likelihood of acceptance. Another approach that results indicate may include taking a relational, social identity and norms approach and delivering such messages from trusted in-group members.

R1: All figures would benefit from significantly more explanatory test, so they can be read and understood without closely reading the paper.

Response: All 4 figures now have titles/captions included within the figure, clearly labeled x and y axes, and explanations included in figure caption below the figure (provided in a figure captions document). A note regarding figure 2. Figure 2 was presented as a pie chart in the initial submission. However, because percentages do not add up to 100% (because each tweet could be coded with more than one code) it is

more appropriate to graphically present the six derived themes as bar charts. Figure 2 has accordingly been changed to be a bar chart.

R1: Figure 4 would be greatly improved if the time-intervals were clarified. There seems to be a surprising periodicity. I can only assume this is because of (uneven) time-period binning. Otherwise, this should be explained.

Response: The time-period binning for figure 4 (i.e., the x-axis time line) is actually even. The data points used for figure 4 are aggregated by 7-day periods (weeks); for each month in the graph, there are 4 data points. Each data point represents the frequency for a certain label during the past 7-day period.

R1 (Methods questions): The low intercoder reliability seems to imply that the concepts and interpretations were unclear even so to the coders. Is there a literature on twitter qualitative analysis that can provide comparative numbers in other studies? Or some reason (other than lack of construct clarity in overlap) to think that this isn't a defect in the analysis? Also, the specific method used seems difficult. It seems some specific source or justification is needed for applying Fleiss's Kappa in this multi-categorical assignment case (a statistician should be consulted).

Response: The intercoder reliability (IRR) computed for the derived themes indicated substantial agreement (>0.6 according to Fleiss Kappa where 0 is no agreement, 1 is perfect agreement, and >0.6 indicates substantial agreement; Zapf et al, 2016; Feng, 2015; Hallgren 2012; Fleiss, 1971) among 3 coders for 6 of the 7 themes. Given that there were 3 coders that needed to come to agreement on the codes (for nominal data), we appropriately used Fleiss Kappa statistic, which is the most rigorous and appropriate approach to estimate IRR among 3 coders for nominal codes that takes chance agreement into account. Cohen's kappa is not appropriate when there are >2 coders (Zapf et al, 2016; Feng, 2015; Hallgren 2012; Fleiss, 1971- the latter 2 references Hallgren and Fleiss were cited in the manuscript in the methods section, page 9, under the heading Intercoder reliability, lines 191-196). The coding team discussed, reviewed and interpreted codes, and coders were rigorously trained throughout 9 months (May 2020-January 2021) on achieving a consistent interpretation of tweets. As a result of iterative discussion, the development of a refined codebook with detailed inclusion and exclusion criteria, and testing IRR on independent and randomly sampled data sets, the coders gained a nuanced and similar understanding and interpretation of the codes. IRR was reported to communicate rigor and transparency and that the analysis was performed conscientiously and consistently.
Social media content analysis IRR reporting varies considerably. The literature shows that reporting %agreement is common however, this is inappropriate as it reflects an inflated agreement since some may be by chance. If two coders are used, then Cohen's kappa is appropriate. If 3 or more coders are used to code nominal data, then Fleiss Kappa or Krippendorf's alpha IRR statistic are appropriate. Fleiss Kappa compares observed agreement with expected agreement while Krippendorf alpha measures observed and expected disagreement.

One of the 7 derived themes, desensitization, had a low intercoder reliability. Fleiss kappa statistic formula penalizes low sample size and when there is low variance on disagreement. After much discussion, we have removed this theme given that the IRR did not indicate substantial agreement on the tweets coded in this dataset. The phenomenon emerged in May 2020 but it was <1% of the mask tweets. We believe that "desensitization" warrants further investigation for future research and note this in the discussion section of the manuscript.

R1 (Methods): The dataset was filtered to remove tweets that only contained hashtags or user mentions. Does this exclude when a reply message is intended to highlight a tweet? Does this exclude quote-tweets with only a hashtag or mention?

Response: The dataset was filtered to remove hashtags and mentions among the 7000 mask tweets to analyze these, but we did not remove those tweets. Table1 describes the types and range of "mentions" while Table 3 describes the evolution in hashtags over time. Furthermore, the dataset we used for coding was user generated content only. In other words, tweets that contained novel text generated by the user. This did not include instances where a user retweeted another tweet with no additional text or commentary. This has been included in the manuscript, page 9, lines 167-168.

R1 (Methods): The keyword list was clearly designed to catch non-English tweets, but then tweets were geolocated and only English tweets were included. This should be explained (presumably data collection is being used for other projects, which is fine, but should be stated).

Response: COVID19 and coronavirus tweets were being collected at large from the beginning of the pandemic (January 2020) for the data to be used for a number of analyses (which included multiple languages and a global analysis). For the focused mask tweet analysis, as a research team we made the decision to narrow the scope to the US with respect to mask behaviors, attitudes, and COVID19 risk perception given that mask wearing was uniquely controversial and political in the US. This has been stated on page 9, lines 152-153.

R1 (Methods): Given the global nature of the risk and spread, it seems strange that discussion of masks in places other than the US should be automatically coded as non-relevant. Can you explain this choice?

Response: While COVID19 and coronavirus tweets were being collected at large from the beginning of the pandemic (January 2020) for the data to be used for a number of analyses (which included multiple languages and global), we/the research team focused our mask tweet analysis on the US, because this topic was controversial and politicized in the US at the time. We did not code other tweets as non-relevant: they were filtered out and not included before we began coding. We filtered the dataset to be focused on U.S. English tweets about masks and mask wearing during the early phases of the pandemic.

R1 MINOR EDITS

R1: Data availability upon request is insufficient, as the guidelines state. Despite twitter terms of service restricting sharing the full dataset publicly, the tweet ids can be made fully public to allow reconstruction of the dataset without requesting data. If this is not done, a clear justification is needed. Also, the availability statement also states that the codebook would be available upon request but it is in fact included as appendix S3.

Response: According to the Twitter agreement at https://developer.twitter.com/en/developer-terms/more-on-restricted-use-cases, we release the IDs (Tweet_ID and User_ID) of the 7K mask tweets, but not their message content. The IDs (Tweet_ID and User_ID) are published in the file at https://github.com/ISG-ICS/Geolocated_USA_tweets_w_mask_IDs/blob/main/geolocated_USA_tweets_w_mask_full%20-%20geolocated_USA_tweets_w_mask_IDs_only.csv.

We have made the codebook available in S3 to be transparent about our data analysis process i.e., the qualitative content data analysis. It is common as part of rigor of qualitative content analysis to make the codebook available as part of an "audit trail" to be able to replicate findings.

R1: Ethics statement should have a review number.

Response: This research qualifies as exempt from IRB review at the University of California Irvine where the study was done. Upon advice from the UCI IRB, they stated that provided that the online data is publicly available via Twitter, it is appropriate to categorize this under Exempt Category 2i for the observation of public behavior (see below). Note that our research fits this criteria.

Research that includes only interactions involving educational tests (cognitive, diagnostic, aptitude, achievement), survey procedures, interview procedures, or observation of public behavior (including visual or auditory recording) [1]
One of the following criteria must be met:<br><br>*2i) The information obtained is recorded by the investigator in such a manner that the identity of the human subjects **CANNOT** readily be ascertained, directly or through identifiers linked to the subjects,*<br><br>*2ii) Any disclosure of the human subjects' responses outside the research would **NOT** reasonably* place the subjects at risk of criminal or civil liability or be damaging to the subjects' financial standing, employability, educational advancement, or reputation*<br><br>**Reasonably defined as with fair and sound judgment; a standard used by an ordinary, rational person under similar circumstances.*

Given the self-determined nature of these processes & their exemption from IRB review, UCI does not issue IRB approval numbers. Please see attached documentation from the UCI IRB website that further outlines the rationale for the exempt self-determination process.

R2: Re: people's risk perception…there are interesting contextual considerations …while some of the conclusions such as the need to build a presence and trust of public health officials seem a bit lacking in novelty, devising messages according to social and group identity of the recipient rather than increasing severity and threat is a new recommendation. I would like to confirm a few points.

1. How much influence does twitter have on what range of people compared to other media?

Response: According to Statista, as of February 2021, 42% of adults in the US between ages 18 and 29 use Twitter. This age group was the microblogging's biggest audience in the US followed by 27% of 30 to 49-year-olds, 18% of 50 to 64 year-olds, followed by 7% of 64+. As of 2019, Twitter had 69 million monthly active users in the US. Thus, the reach among the young adult and adult US population is substantial with reach and daily exposure. Furthermore, as of January 2021, 61.6% of Twitter users were male, and 38.4% female. As of 2020, Twitter was the most popular social network for news consumption. Twitter is popular among users looking to catch up and contribute to current and trending topics and to live-tweet about events and media. According to Pew Research Center (2019), Twitter users are more likely to be younger and Democrat than the general public. An analysis by Pew Research Center also showed that 22% of American adults who use Twitter are representative of the broader population in some key ways but not others. They are more likely to identify as Democrat, be highly educated, and have higher incomes. However, their views are diverse on a range of topics. Relative to other social media platforms, Twitter is the 13th most used social media platform, with 397 million active users relative to for example Facebook, which is the most popular at 2,830 million users. This has been discussed in the limitations section.

2. Does the difference in expertise of the three coders affect the results of the analysis? It would be better to clarify the background of coders.

Response: The three coders, a public health doctoral student, a senior psychology undergraduate student and a graduate social computing/HCI (human computer interactions) student were trained extensively, and coded the tweets independently. They met 3x weekly (M,W,F) with PI (Hopfer) across 9 months (May 2020-January2021), discussed the labeling and interpretation of codes weekly in an iterative manner to arrive at consistent interpretation and coding, refined development of a codebook with detailed exclusion and inclusion criteria, and reached a minimum of 0.6 Kappa statistic on each of the derived codes with the exception of the "desensitization" code, which we have now removed.

3. Although the overall appearance rate of COVID-19 desensitization is low, why was it extracted as one category and the kappa coefficient remained low at 0.28?

Response: *The code about COVID19 desensitization was observed in the data beginning in May 2020 and beyond (data went through July 7, 2020) although relative to other mask tweets the numbers were few. Qualitatively, the phenomenon was observed in the data (e.g.,* a sample Tweet is as follows: "*It seems that we no longer care about the #pandemic. #coronavirus scare was a whole 3 months ago and we're tired of wearing masks and practicing social distancing*", June 26th). *Of the ~7000 mask tweets, there were relatively fewer tweets (~70) about COVID19 and mask wearing fatigue with some of the tweets overlapping with other codes ('mask behavior of others' and 'who is at risk'). We initially included this code in the paper because the purpose of a qualitative content analysis is to discover qualitatively relevant and emerging codes related to the phenomena of mask wearing and COVID19 risk perception (not necessarily determined by frequency). However, in light of the poor IRR agreement we have removed this code and this topic will warrant future research.*

4. How does tailoring public health messaging by psycho-behavioral profiles to effectively reach heterogenous subgroups regarding covid-19 risk in the discussion specifically refer to?

Response: In the discussion section, in particular, the "Implications for public health messaging" section, we have revised this section of the manuscript. Results of the content analysis indicate that social identity and structural determinants of risk, in addition to perceived severity and threat, shape COVID-19 risk perception. With the "mask behavior of others" being the most frequent way in which masking was discussed that reflected COVID-19 perceived risk (and several additional themes e.g., politicization, reflecting social experiences of risk), one messaging approach may include relational and normative messaging delivered by in-group members. The sentence about tailoring was removed for clarity.

R4. Authors' analysis of the social dynamics of risk perceptions regarding mask wearing in the COVID-19 era seem to represent the history of American society's reaction to COVID-19. This study allows us to think about how risk perception is diffused in a crisis, which could have important findings for determining public health strategies. A few concerns remain:

1. We defined risk perception as a communication process situating risk perception cognitively. Your analysis did not address the features of Twitter's communicable tools, such as mentions, retweets, and replies, and thus, did not adequately discuss the relationships within and between the seven themes. Is it possible the results represent the size of the reaction to social events rather than describing risk perception as a communication process? Add a discussion of the communication process within or across themes with the limitations of this study's analysis.

Response: A limitation of the study includes that we did not additionally analyze the functional communication properties of social media (e.g., hashtags, hyperlinks, mentions in detail, retweets – all of which can serve to amplify or attenuate messages among networks). We did however, qualitatively describe the evolution of hashtags (Table 3), the types of "mentions" (Table 1 and reported the proportion of tweets that used these strategies along with hyperlinks). We were not able to report on use of retweets since this was absent in our data. All of our data was user generated, original tweets as mentioned in the methods section. Examining the amplification or attenuation of the functional social media communication properties (hyperlinks, hashtags, mentions, etc) is outside the scope of this content analysis and warrants further research. We mention this in the limitation section.
We add a discussion of the communication process within or across themes with the limitations of this study's analysis on page 39. Hyperlinks likely further contextualize comments, while mentions and hashtags likely amplify or attenuate depending on the nature of the hashtag and the individual being mentioned.

2. The process of risk perception includes intensify and attenuate perceptions. Figure 4 shows that the tweets for each risk perception theme repeatedly increase and decrease. However, your discussion was biased towards an increase in risk perception. It would be better if you also discussed attenuating risk perception. These comments may contain my misunderstanding. If so, please point it out.

Response: Thank for you this comment and observation. We discuss both the intensification i.e., amplification and attenuation of messages. In the discussion section, beginning with line 605, we mention the amplification and attenuation of mask tweets (page 28), followed by lines 612 discusses the intensification of severity and lines 614-615 the downplaying of severity with comparisons to flu e.g. (page 28), lines 624 and 632 discuss the attenuation of risk through mask effectiveness debates (page 29), lines 638 mentions both amplification and attenuation of people sharing personal experiences with COVID19 (top of page 30), line 688 mentions amplification of risk through the use of mentions (page 32), line 701 (page 32) speaks to the attenuation of risk perceptions as a result of politicization, line 718 (page 33) describes the intensification of COVID19 risk perception early on with mask wearing observed in public settings for the first time and when it was not yet normative to wear masks outside of medical settings; line 743 mentions amplification and attenuation in the heading (also title of paper was changed to acknowledge both); line 752 acknowledges amplification and attenuation both occurring simultaneously; lines 754-759, line 771 discuss amplification and attenuation (page 35);line 775 (page 36) acknowledges the simultaneous amplification and attenuation of risk occurring; lines 794 (page 36).

Sincerely,

Suellen Hopfer
Suellen Hopfer
